# Satellite tagging of rehabilitated green sea turtles *Chelonia mydas* from the United Arab Emirates, including the longest tracked journey for the species

**DOI:** 10.1371/journal.pone.0184286

**Published:** 2017-09-05

**Authors:** David P. Robinson, Rima W. Jabado, Christoph A. Rohner, Simon J. Pierce, Kevin P. Hyland, Warren R. Baverstock

**Affiliations:** 1 Jumeirah Group, Dubai, United Arab Emirates; 2 Gulf Elasmo Project, Dubai, United Arab Emirates; 3 Marine Megafauna Foundation, Truckee, California, United States of America; 4 Wildlife Protection Office, Dubai, United Arab Emirates; Deakin University, AUSTRALIA

## Abstract

We collected movement data for eight rehabilitated and satellite-tagged green sea turtles *Chelonia mydas* released off the United Arab Emirates between 2005 and 2013. Rehabilitation periods ranged from 96 to 1353 days (mean = 437 ± 399 days). Seven of the eight tagged turtles survived after release; one turtle was killed by what is thought to be a post-release spear gun wound. The majority of turtles (63%) used shallow-water core habitats and established home ranges between Dubai and Abu Dhabi, the same area in which they had originally washed ashore prior to rescue. Four turtles made movements across international boundaries, highlighting that regional cooperation is necessary for the management of the species. One turtle swam from Fujairah to the Andaman Sea, a total distance of 8283 km, which is the longest published track of a green turtle. This study demonstrates that sea turtles can be successfully reintroduced into the wild after sustaining serious injury and undergoing prolonged periods of intense rehabilitation.

## Introduction

The green sea turtle *Chelonia mydas* (Linnaeus 1758) is found circumglobally in tropical and subtropical waters. It is a highly migratory, long-lived species that is susceptible to anthropogenic pressures during all life-stages [1]. Green turtle populations around the world are declining due to harvesting for meat and eggs [2–6], incidental capture in both artisanal and commercial fisheries [7,8], as well as habitat alteration, degradation and loss [9,10]. Despite ongoing worldwide protection efforts spanning several decades, green turtles remain classified as Endangered on the International Union for the Conservation of Nature (IUCN) Red List of Threatened Species [1]. Furthermore, it has recently been suggested that traditional approaches to estimation of numbers of nesting turtles may have led to a gross overestimation by as much as 50%, further highlighting the ‘endangered’ status of this species [11]. On the other hand, there have been a number of very encouraging conservation successes suggesting upward trends in population sizes (e.g. Ascension Islands, [12]) and showcasing that reversing declining trends in populations is possible.

The Arabian Gulf (hereby referred to as ‘the Gulf’) is located within a subtropical, hyper-arid region. The Gulf is a shallow basin, bordered by Bahrain, Iran, Iraq, Kuwait, Oman, Qatar, Saudi Arabia and the United Arab Emirates (UAE).

Within the Gulf, the green turtle has nesting sites in Saudi Arabia [13–15], Kuwait [16–18], Iran [19], and the UAE [20]. The region is undergoing rapid economic growth, involving substantial construction in coastal and offshore regions [21]. The Gulf is the warmest sea in the world [21] and environmental conditions within the Arabian Gulf are among the most extreme on the planet [22].Although nesting sites have been monitored for many years, information on the movement ecology of green turtles in Gulf waters is limited to a single study that used satellite tracking in the northern waters of Kuwait [18]. Given that green turtles spend the majority of their lives away from nesting sites, understanding their movements and habitat use is essential to ensure appropriate management and protection for this Endangered species [18,23].

Advances in satellite tracking technologies have led to improved insight into the life history and behavior of many species [24,25] and facilitated the identification of critical habitats to support management actions (e.g. [6,26–28]). For example, such studies have shown that green turtles migrate from feeding grounds in Brazil to nesting grounds in the Ascension islands, a distance of at least 2300 km [29–31]. Hays et al. [32] tracked eight green turtles from the Chagos Archipelago and found that seven of them made journeys exceeding 1000km to their foraging grounds.

Gilbert [33] states that the main objective of rehabilitating sea turtles is to contribute to their conservation. Rehabilitation is particularly beneficial for sea turtles as they have a low survival probability from egg to maturity; the rehabilitation of individuals that have survived the high initial mortality rate is therefore considered demographically relevant [34]. However, there is currently a gap in our understanding of post-rehabilitation integration back into the wild and survival rate, with mixed results reported in the literature. Few studies have focused on post-release behavior of rehabilitated turtles [33,34] with most turtle satellite tracking work focusing on the post-nesting movements of adult females [25,35]. Godley et al. [35] suggest that satellite tracking is a valid way of monitoring post-rehabilitation and release survival, and that more information needs to be gathered on male and immature turtles. However, they question the normality of the rehabilitated turtles’ post-release behaviour. Cardona et al. [36] highlight the importance of understanding the ability of rehabilitated sea turtles to survive and integrate back into the reproductive population successfully, not only to justify rehabilitation efforts and costs, but also because rehabilitation centres often supply animals for satellite tracking studies [37,38]. Mestre et al. [34] state that post-release tracking of rehabilitated sea turtles is a valid means to monitor the success of the rehabilitation process, while obtaining additional information on their movements and behaviour.

Satellite tracking of rehabilitated turtles has produced mixed reports with regards to behavior and integration back into the wild. Cardona et al. [36] satellite tracked 18 loggerhead *Caretta caretta* (Linnaeus, 1758) turtles off Spain, six of which had undergone ‘long and complicated’ rehabilitation protocols. In a comparison to 12 control turtles, it was found that two of the six rehabilitated animals did not differ from the control animals, but four animals showed anomalies in one of the tested behavioral parameters. They concluded that rehabilitated turtles survive in the wild for at least a few months, but questioned their long-term re-adaptation to natural conditions. Polovina et al. [39] found that satellite-tagged captive-raised loggerhead turtles in Japan did not differ from wild individuals in their dispersal patterns. Nichols et al. [40] reported that a loggerhead turtle, shown to be of Japanese origin through genetic analysis but captured from the wild as a juvenile off the Pacific coast of Mexico, migrated back to Japan from Mexico after 10 years in captivity. These studies generally indicate that periods of captivity are unlikely to lead to major differences in post-release foraging or navigation [36]. Rees et al. [18] tracked three green turtles in the Gulf, two of which were rehabilitated animals, and found these turtles moved to foraging sites and developed similar home ranges, suggesting survival and successful re-integration of the rehabilitated animals. In addition, a large number of rehabilitated sea turtles have been satellite-tracked from Naples, Italy and, these animals were used to investigate diving behaviour and seasonal changes [38,41–43]. Mestre et al. [34] satellite tagged two green and one loggerhead turtle after long periods of captivity (up to 30 years) off Portugal. These turtles moved towards known feeding locations for both species, suggesting successful reintegration into the wild.

The Dubai Turtle Rehabilitation Project (DTRP), based in the UAE, has been running in its current form since 2004 and receives and treats sick or injured turtles from countries surrounding the Gulf [44]. Satellite-tagging of rehabilitated green turtles by the DTRP has been ongoing since 2005. Here we present data on the spatial ecology, post-release behavior and thermal environment data of eight rescued and rehabilitated sub-adult and adult green turtles released after varying periods in captivity.

## Materials and methods

### Study area

The Gulf is a shallow, almost enclosed sea experiencing low precipitation and high evaporation rates which lead to salinities reaching over 39 ppt. The average depth is 30 m, gradually becoming deeper to 100 m as it approaches its entrance at the narrow Strait of Hormuz [45–47]. Air temperatures in the region can drop to 0°C in the winter and reach in excess of 50°C in the summer, resulting in fluctuations in nearshore waters of up to 29°C over the year, from 10°C in winter to 39°C in summer. Deeper waters vary between 18°C and 33°C [48]. Despite these physical extremes, the Gulf is a highly productive and diverse basin, supporting important habitats such as seagrass beds, mangroves, salt marshes and coral reefs [45,48]. The UAE lies along the southeastern coast of the Gulf, and has two separate coastal areas: the low, sandy Gulf coast, and the rocky and somewhat steep Gulf of Oman coast [49].

### Study animals

Permissions for sea turtle rehabilitation work were given by the Dubai Wildlife Protection Office with whom this work was conducted. Eight green turtles *C*. *mydas* were brought to the DTRP between 2004 and 2011 for rehabilitation from debilitation or infection resulting from ‘cold stunning’ [50] in the winter months (n = 4); severe injuries (n = 3) thought to be caused by propeller impact; intentional anthropogenic injuries or entanglement; and intestinal impaction (n = 1) (Table 1).

**Table 1:** Summary of rehabilitation information and resulting tracking data for green sea turtles *C*. *mydas* released in UAE waters. Minimum bounding geometry (MBG) and percentage volume contours (PVC) are provided for turtles that remained in the Gulf.

Turtle Name	Transient /Resident	Sex / Life Stage	Ailment on receipt	Rehab Duration (days)	Release Weight (kg)	Curved Carapace Length (cm)	Maritime Boundaries Entered	Tracking Duration (days)	Distance traveled (km)	MBG (km^2^)	PVC 95% (km^2^)	PVC 50% (km^2^)
**Dibba**	Transient	Female / Adult	Injury	546	80	93	■ UAE ■ Oman ■ Maldives ■ Sri Lanka ■ India	259	8283	NA	NA	NA
**Maju**	Transient	Unknown / Sub-Adult	Infection	96	25	56	■ UAE ■ Iran ■ Pakistan	47	992	NA	NA	NA
**Lepi**	Transient	Unknown / Sub-Adult	Injury	499	25	57	■ UAE ■ Iran	284	1123	16382	657	69
**Jade**	Resident	Female / Adult	Infection	275	150	115	■ UAE ■ Iran	310	2450	18057	568	8
**Moonlight**	Resident	Unknown / Sub-Adult	Infection	203	32	58	■ UAE	141	190	2564	709	56
**Emerald**	Resident	Unknown / Sub-Adult	Injury	1353	37	70	■ UAE	220	1086	1591	529	61
**Bahar**	Resident	Unknown / Sub-Adult	Infection	263	36	70	■ UAE	52	853	9635	5023	112
**Belle**	Resident	Female / Adult	Impaction	262	58	90	■ UAE	112	9	1813	854	15
**Mean for resident turtles**										8340 **±** 7514	2154 **±** 1783	29 **±** 38

Adult sea turtles are sexually dimorphic, with mature males possessing a long prehensile tail [51]. After internal examination, tail length is the main secondary sexual characteristic of adult sea turtles and has been routinely used to successfully determine the sex of mature animals [52]. Miller (2011) summarized the morphometrics of nesting turtles from records of 2,844 nesting green turtles examined along the Saudi Arabian Gulf coast and found that the mean curved carapace length (CCL) of these adult females ranged between 73–114cm CCL. Rees et al. [18] recorded a CCL of 98.2 and 96cm for nesting females satellite tagged in Kuwait. Our study animals were classified using their CCL and tail length as either sub-adult (<70 cm) or adult (>90 cm) females and ranged in weight from 25 to 150 kg. The adult turtles (n = 3) were all female based on their short tails. Individuals deemed immature could not be sexed.

### Rehabilitation and release

Turtles in this study underwent rehabilitation for 96 to 1353 days (mean = 437 **±** 399 days). Individual rehabilitation protocols were determined by the ailment or injuries suffered, but common treatments while in captivity included rehydration, force-feeding, antibiotic treatment, vitamin and mineral treatment, anti-parasite treatment for debilitation [44], and surgical procedures as detailed in [53] and [54].

All animals were moved into a sea-fed outdoor enclosure and observed for at least two months prior to release. Turtles were evaluated for active feeding and swimming ability, and given a physical health assessment to ensure they met the criteria for release described in [55]. A blood sample was also taken from each turtle and tested for normal parameters using methods and tests reported in [44]. Each turtle was considered fit by all these standards at their time of release.

### Tag deployment

Turtles were released and tracked between January 2005 and April 2013. Sirtrack tags (n = 4) were used until 2010, after which four Wildlife Computers SPOT5 back-mount tags were used. Both tag models estimated location through the Argos satellite system operated by CLS Argos (http://www.argos-system.org). Tags were attached to the carapace using a base of marine putty and covered with a slow-hardening, low temperature-generating epoxy. Once the putty and epoxy were hard, the tags and exposed epoxy were painted with a black copper-based antifouling paint produced by Interlux, leaving a non-painted distance of 1 cm around the tag’s saltwater switches.

All tags had a 45 second Argos transmission repetition period with a limit of 250 transmissions per day. Sirtrack tags were programmed by the manufacturer and duty cycled to transmit at sunrise through to sunset for UAE local time. Deployed Sirtrack tags were not capable of collecting information on temperature. Wildlife Computers SPOT5 tags were programmed using the manufacturer-provided software and duty cycled to transmit from sunrise to sunset for UAE local time. Each Wildlife Computers tag was programmed to record 12 temperature bins ranging from 9°C to 39°C in 3°C increments and form two sets of 12-hour histogram data, one for local daylight hours and one during the hours of darkness. All temperatures above 39°C were included in the final temperature bin, while all temperatures below 12°C were included in the first bin.

### Satellite-data filtering and analysis

Tags included an Argos transmitter and used standard Doppler-based geolocation to track the position of the turtle. An accuracy estimate was assigned and a location class was then given by the Argos system (A, B, 0, 1, 2, 3). Class A and B are not given an error estimate by the Argos system, however, class 0, 1, 2 and 3 have an estimated accuracy of >1500 m, >1000 m, >500 m, >150 m, respectively. To facilitate regular data downloads from the Argos system, accounts were set up through the www.seaturtle.org Satellite Tracking and Analysis Tool (STAT). The STAT automatically collected daily Argos data and stored these data online. Data transmitted by each satellite tag were checked for multiple daily and maximum transmission records and static temperature data that could be indicative of a floating tag. The STAT bathymetry data were then used to calculate the distance travelled between transmission points and to extrapolate the water depth at the location of tag transmission.

The Douglas filter was applied to the data in Movebank (www.movebank.org) to further improve the accuracy of location data [56]. This filter is based on a Maximum Redundant Distance (MRD) to remove unrealistic locations and in addition a Distance, Angle and Rate (DAR) filter was used. The MRD filter retains locations that are near-consecutive within a defined distance threshold [56]. All Argos locations B grade and more accurate were included in the analysis with locations given an accuracy estimate of 1, 2 and 3 always retained. Locations with duplicate timestamps were removed. A MRD radius of 10 km was set along with a maximum realistic rate of movement was set at 5km/h as recommended in [57]. A turning angle filter of 25° was also used to remove any extreme changes in direction as in [18,58–61]. A mean of 5 **±** 4% of outlying data were removed by the filter (n = 8; median = 5%; range 1 – 13%).

### Home range analysis

All filtered tag locations were input to ArcGIS 10.2.1 from Movebank. The “kernel density tool” was used to calculate percentile kernels of location density. Percentage Volume Contours (PVC) representing 50% and 95% of locations were produced in ArcGIS 10.2.1 from the previously produced kernels, using the “Spatial Analyst” and “Reclassify tool” and, following methodology outlined in [62]. The “map algebra” and “raster calculator” tools in ArcGIS 10.2.1 were used to investigate how many individuals used a specific location within their home ranges as described in [62]. Turtles were categorised as transient if they moved out of UAE waters for 10 or more days, or resident if they stayed within the UAE’s maritime boundaries. Part of the final home range of one turtle (‘Bahar’) was been excluded from these analyses since this individual was recovered from the western region of the UAE after washing up on the shore with a fatal injury thought to be from a speargun.

### Temperature

Water temperature was collected daily in the early morning by internal thermometer in front of the extraction point from the Dubai Madinat Jumeirah coastal water pumps, located ~100 m from the shoreline and intake, from a depth of ~10 m. To investigate temperature related movements in coastal locations, a linear regression was fitted to daily coastal water temperature data and water depth at transmission locations (n = 327) for all five resident green turtles.

## Results

### Tracking

Tracking duration ranged between 47 and 310 days with a mean of 178 **±** 104 days (median = 181, n = 8) and distances travelled ranged between 190 and 8283 km with a mean of 1988 **±** 926 km (median = 1039, n = 8) (Table 1). Minimum bounding geometry for turtles that remained within the Gulf ranged between 1591 and 18057 km^2^ with a mean of 8340 **±** 7514 (median = 6100, n = 6). For approximately three days’ post-release, turtles usually travelled slowly or stayed offshore of the release point. After this period, most turtles moved away in an increasingly focused direction.

### Horizontal movements

Seven turtles were recovered from the west coast of the UAE and released back into the same body of water (Gulf) while one turtle was recovered from, and released on, the east coast (Gulf of Oman). Five turtles remained resident in UAE waters over the duration of tracking. Four of these, ‘Emerald’, ‘Belle’, ‘Bahar’ and ‘Moonlight’, chose core habitats on the coast of the UAE between Abu Dhabi and Dubai and did not leave the area.

The home range area for resident turtles ranged between 529 and 5023 km^2^ with a mean of 1536 **±** 1953 km^2^ (median = 709, n = 5). The core habitat area for resident turtles ranged between 8 km^2^ and 112 km^2^ with a mean of 50 **±** 42 km^2^ (median = 56, n = 5). Transient animals did not form a definitive home range or core habitat area. Both transient (197 **±** 130, median = 259 days, n = 3) and resident (167 **±** 100, median = 141 days, n = 5) turtles had a similar mean tracking duration.

**Fig 1:**
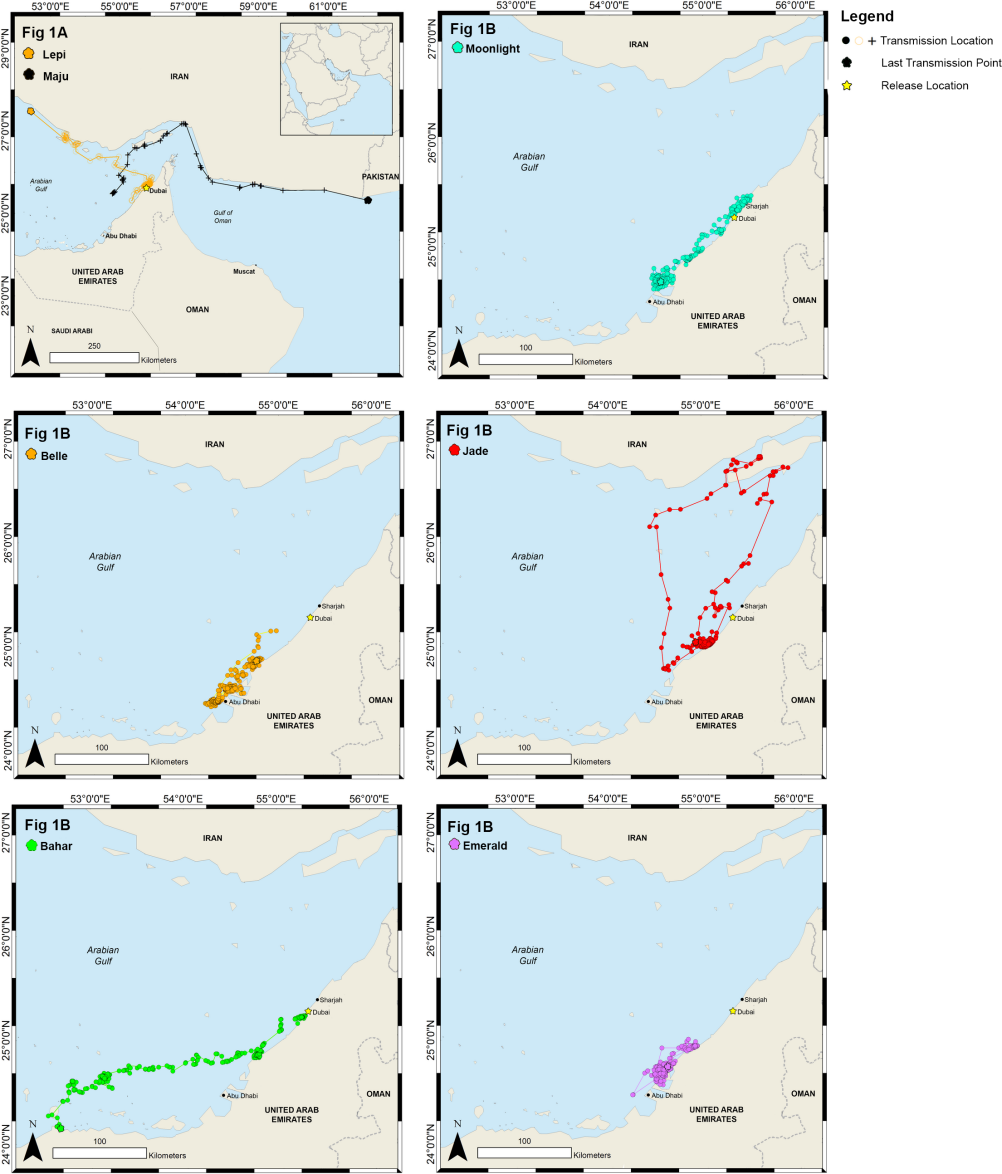
Horizontal movements of seven rehabilitated green sea turtles satellite tagged and released in Dubai, UAE. Fig 1A shows the tracks for ‘transient’ turtles and Fig 1B shows the tracks for turtles considered ‘resident’.

The female adult turtle, ‘Jade’, also formed a small core habitat (8 km^2^) in the Ghantoot area. During her tracking, she made a highly directional journey across the Gulf to Qeshm Island in Iranian waters (Fig 1B). The crossing took 11 days, following which she spent 14 days around Qeshm Island. She then travelled back to her previous small core habitat area over a period of 10 days. Tracking ended 18 days after Jade’s return to her Ghantoot core habitat area. This journey resulted in a large minimum bounding geometry (18057 km^2^) and home range (568 km^2^) for this individual.

Sub-adults ‘Lepi’ and ‘Maju’ were transient: ‘Lepi’ crossed the Gulf, spending a significant amount of time in Iranian waters, while ‘Maju’ crossed the Gulf into Iranian waters, then travelled through the Strait of Hormuz and out into the Gulf of Oman. ‘Maju’ remained close to the coastline, and the last transmission was made just after crossing the maritime boundary into Pakistani waters (Fig 1A).

### Dibba

**Fig 2:**
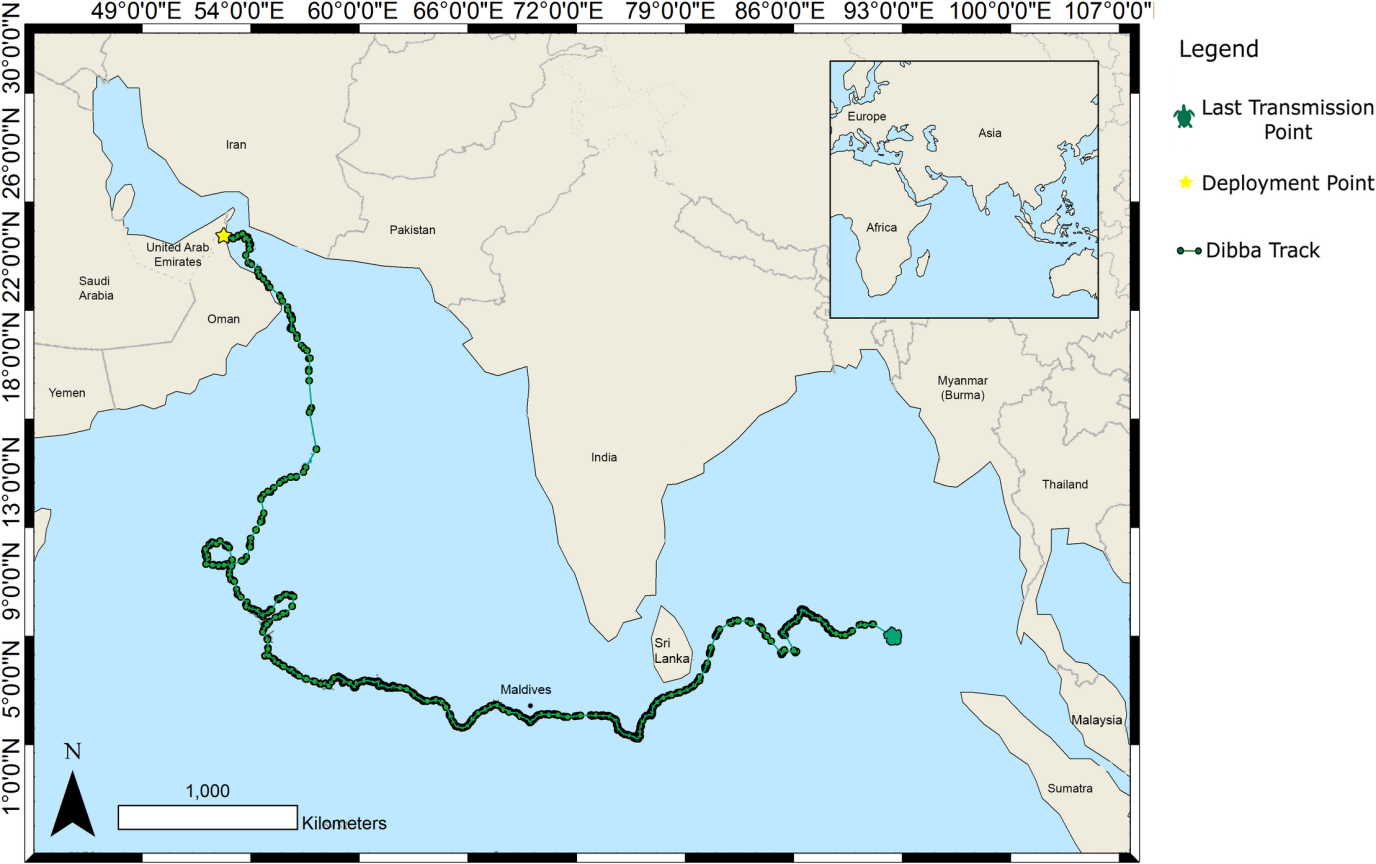
Horizontal movements of ‘Dibba’, a rehabilitated green sea turtle released on the east coast of the UAE in February 2008.

‘Dibba’ was found on the east coast of the UAE in August 2006 with massive head trauma, believed to be intentional and anthropogenic in origin, and was close to death upon arrival at the DTRP. Her rehabilitation took 546 days with treatment given for both the original injury and subsequent illnesses associated with her head trauma. After being monitored for several months in a large sea-fed enclosure, ‘Dibba’ was released close to the area where she was found in February 2008. ‘Dibba’ travelled 8283 km from the UAE, to Omani waters, crossing the Arabian Sea to the Maldives, before proceeding to Sri Lanka and entering the Bay of Bengal where the last transmission was made close to the Andaman and Nicobar Islands (Fig 2). Tag transmissions were not received on a consistent daily basis, message numbers were inconsistent and, the location accuracies of the Argos plots varied throughout the deployment indicating that the turtle was healthy, making dives and travelling independently. The tag is thought to have stopped transmitting at the end of the deployment due to the battery running out.

### Seasonal habitat usage

**Fig 3:**
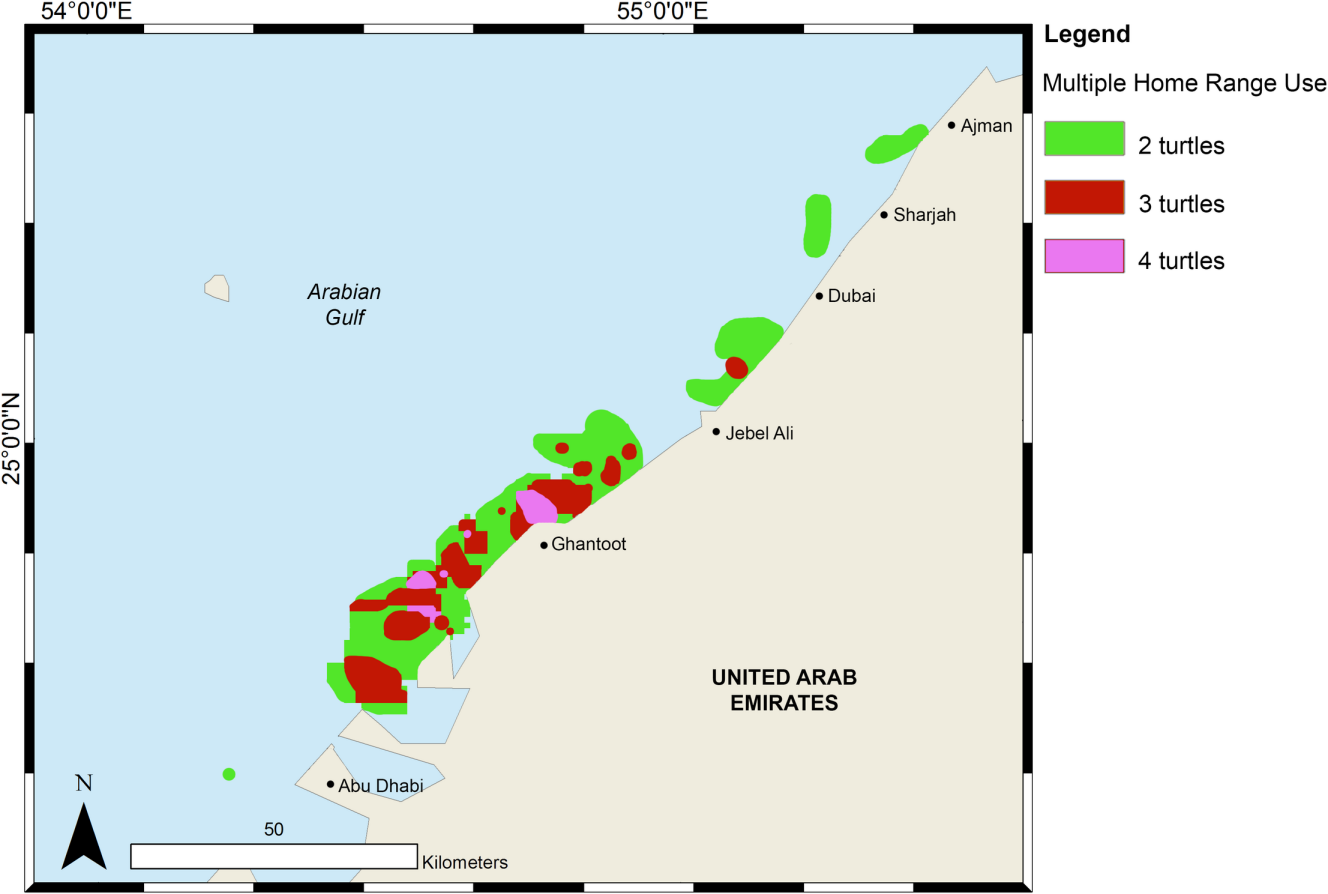
Overlapping home range usage for seven green sea turtles within the United Arab Emirates.

Green turtles used most of the coastline from Abu Dhabi through to Ras Al Khaimah within the UAE (Fig 3). Overlapping core habitats of two or more turtles occurred offshore of Abu Dhabi and Jebel Ali. The Ghantoot area was utilised heavily with three core home ranges overlapping.

Turtles were released at different times throughout the year and had different data transmission periods. Both coastal water temperature (F-statistic: 0.5226 on 1 and 325 DF, p-value = 0.4703) and sea surface temperature (F-statistic: 0.066 on 1 and 325 DF, p-value = 0.80) had no influence on the sea floor depth where turtles made transmissions, showing that resident green sea turtles made no temperature related movements to deeper water.

**Fig 4:**
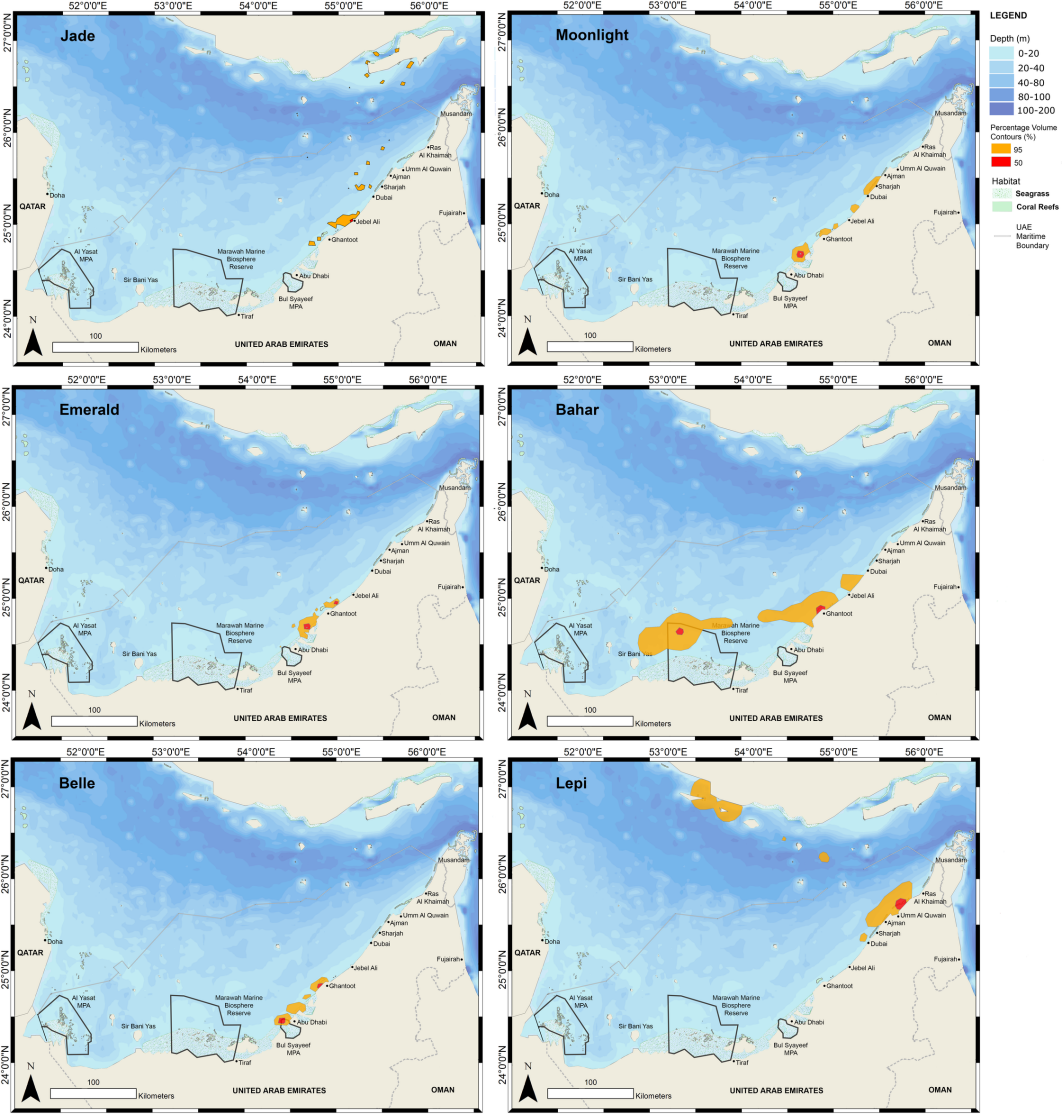
Fifty percent (core habitat) and 95% percentage volume contours (PVC) for all turtles that remained within the Gulf.

All core habitats of resident turtles were in coastal, shallow waters within known seagrass areas (Fig 4). The only core habitat of a turtle that was not immediately coastal, although still in water less than 10 m in depth, was observed in ‘Bahar’. This turtle’s core habitat included two separate areas, one immediately coastal and one area approximately 50 km from the coastline (Fig 4). The core habitats of two turtles, ‘Bahar’ and ‘Belle’ fell within current protected areas, although at the time of tagging the Bul Syayeef Marine Protected Area (MPA) had not yet been declared.

### Tag-recorded temperatures

**Fig 5:**
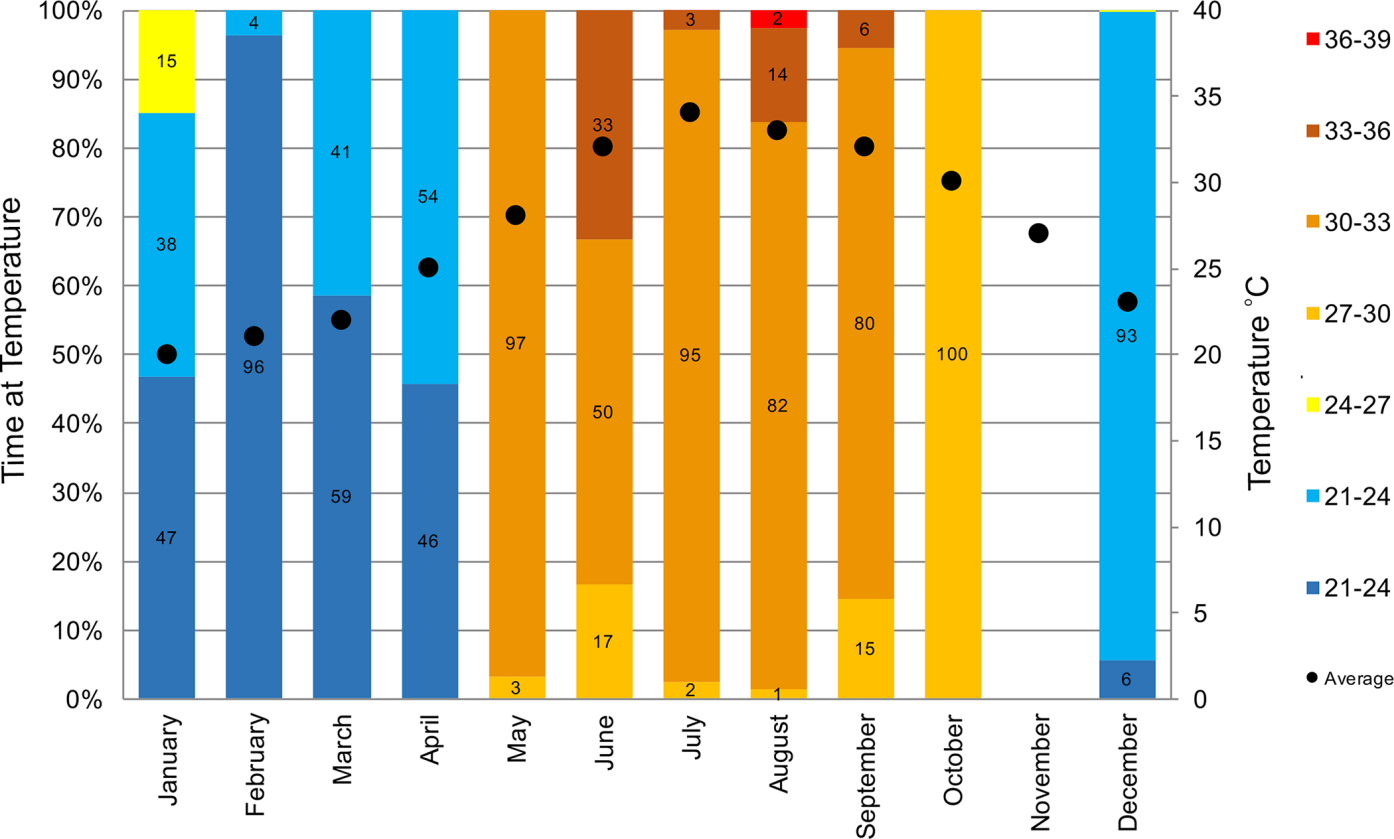
Overall percentage of time spent at temperature for all SPOT tagged turtles, split into months, compared to average monthly water temperatures taken in coastal Dubai at 10 m depth (black dots). No tags were active during the month of November.

No notable thermoregulatory movements were noted during summer. The major temperature bin recorded by turtle-borne tags was similar to the mean water temperatures recorded daily at 10 m depth in Dubai. The highest temperatures experienced by these turtles varied between the 36–39°C data bin in August and the 21–24°C temperature bin in the cooler winter months (December to April). In summer, the prevalent temperature bin experienced was between 30–33°C, although time was spent in each bin. In winter, turtles experienced the normal range of temperatures associated with coastal waters, from 20°C to 34°C (Fig 5).

## Discussion

### Post-release movements

All eight green turtles *C*. *mydas* included in this study showed a successful integration back into the wild after long periods of rehabilitation. This builds on the successful rehabilitation efforts documented in previous studies from other areas [18,33,34]. Turtles in this study returned to either known feeding areas or undertook large-scale migrations, indicating they were fit to resume normal behaviours. Rehabilitation efforts thus were a direct benefit to these individuals, contributing to the survival of this endangered species and thereby supporting conservation efforts. The associated post-release tracking also provided information on the lack of obvious thermoregulatory behavior of green turtles in an water body subject to extreme water temperature variation.

There are limited previous satellite-tagging data on the movement of green turtles in the Arabian region. Rees et al. [58] satellite-tagged two nesting adult green turtles from the Island of Masirah, Oman that travelled into the Red Sea. Journeys were reported to be a minimum of 2400 km and 2500 km, respectively, crossing maritime boundaries including Yemen, Eritrea and the Red Sea coast of Saudi Arabia. Rees et al. [18] tracked three adult females from Kuwaiti waters of the Gulf, one post-nesting and two rehabilitated. The rehabilitated turtles displayed no notable difference in post-tagging behavior when compared to the nesting female. Unlike studies focused on nesting females, rehabilitated turtles are typically from unknown origin, with no data on their birthplace, nesting location and migration routes. The turtles included in this study spanned a range of ages, injuries, and ailments. This could explain why some of the animals tagged were transient to UAE waters, whereas others remained ‘resident’. Transient turtles could have been injured while moving through UAE waters on a larger-scale migration, which they resumed after successful rehabilitation.

‘Jade’ was the only resident turtle to leave UAE waters for a short period of time and then return to her previous core habitat foraging area. On July 25 2010, ‘Jade’ made a directional movement across the Gulf to Qeshm Island, and then returned to her preferred core habitat area. This movement was highly directional, and could possibly represent movement towards a nesting area or a quest to find a mate. Spotila [63] reported that green turtles lay up to three nests in a season and have an inter-nesting interval of 12–14 days. ‘Jade’s’ CCL on release was 115 cm, indicating she was mature, and she spent 14 days around Qeshm. This could have been sufficient time to lay two batches of eggs before returning to her foraging habitat around Ghantoot. Although there are no previous reports of green turtles nesting on Qeshm Island, Jade’s movement was during the summer months of July and August when green turtles nest within Gulf waters [18]. Nesting could not be confirmed through temperature or ‘dry’ time as the tag used was not capable of collecting temperature or ‘haul out’ information. However, movement for foraging was unlikely due to the long transit times (11 days to Qeshm and 10 days on the return to Ghantoot) and the known productivity of her core habitat.

‘Dibba’ was the only turtle to be found and released on the east coast of the UAE. The longest reported post-nesting movement by an adult green turtle is from the Chagos Archipelago to mainland Africa, a distance of 3979 km [64]. ‘Dibba’ moved more than twice this distance travelling 8283 km and was still travelling when the tag stopped transmitting. This journey is comparable to the trans-oceanic movements known to be made by loggerhead turtles migrating across the Pacific Ocean at different stages of their life [40].

This highlights the exceptional abilities of green turtles to travel and navigate over long distances across large expanses of open ocean. Karl & Bowen [65] conducted a global study on green turtles from 15 major rookeries around the world, categorising haplotypes into two groups corresponding to major oceanic basins: the Atlantic and the Indo-Pacific. The latter included turtles sampled from the Ras Al Hadd rookery in Oman. They demonstrated that there was population substructure within each basin, suggesting natal homing by female green turtles, but they also noted haplotype connection among the Indian and Pacific Ocean rookeries suggesting some degree of interchange. Although ‘Dibba’ was found and released on the east coast of the UAE, and not in the Gulf itself, Dibba’s journey away from the region may not be unusual and we hypothesize that it is unrelated to her previous injuries and rehabilitation. Aloia & Al-Ghais [66] investigated population genetics of foraging green turtles in the Gulf and mtDNA results suggested that at least two populations occur in this area. They proposed that these turtles may be from nesting and wintering populations and could have travelled from as far as the Seychelles, Pakistan, India or the Maldives. We show here that green turtles leave the Gulf, and it is equally possible that they move into the Gulf from Omani waters [67]. Carr [30] stated that the occurrence of wandering and straying must happen and that such occurrences are advantageous for green turtle colonies. We suggest that further study into the regional movements and population genetics of green turtles should be conducted to expand on our knowledge of their movements from this region.

### Post-release residency in foraging areas

Our results show that sub-adult and adult green turtles use the same near-shore habitat, with some home ranges directly overlapping. Luschi et al. [68] suggested that sub-adults of most turtle species recruit to neritic habitats after some years of pelagic life to complete their development. The authors highlighted that these areas are either shared with adults (who use them as foraging grounds) or can be frequented only by sub-adults that later shift to a different adult feeding area [69]. Another study by [70], where two sub-adult green turtles were tagged after rehabilitation, found that the turtles remained close to the release site. They suggested that these turtles did not need to move far because of the habitat suitability around the release site. Similarly, [34] indicated that two green turtles tagged after rehabilitation undertook coastal movements as an optimum strategy to increase their chances of finding food. Since all sub-adult turtles in our study were transient, it is likely they were still undertaking their developmental migrations and heading towards their late developmental habitats, which are likely to be closer to their natal beaches [68].

Adults in our study were residents and utilised most of the coastal region of the UAE from Ras Al Khaimah to Abu Dhabi waters. Overlapping areas, where two or more turtles were present, were mostly located south of Dubai and north of Abu Dhabi with Ghantoot being the most frequented. Ghantoot is a small area that encompasses a large expanse of seagrass beds where foraging green turtles are frequently sighted (RWJ pers. obs). Upon leaving a nesting area, adult turtles usually swim towards a fixed feeding area, generally a neritic environment, where they remain for a long time, possibly for the entire inter-reproductive period [68]. A similar behavior was noted by [18] where rehabilitated green turtles in Kuwait established long-term residencies around the areas they were released and were believed to be foraging there. Although females were resident in UAE waters, they are not known to nest in these coastal areas. They are therefore using these habitats during their inter-reproductive period and then presumably move to other areas within or outside the Gulf to nest. Gaining a better understanding of the use of this foraging habitat is needed to ensure targeted management actions can be developed.

Home ranges of turtles utilising neritic foraging habitats are not well understood [71]. Aloia & Al-Ghais [66] proposed that Gulf green turtles are from two genetic populations, ‘nesting’ and ‘wintering’, and could have travelled from the wider Indian Ocean. By tagging both ‘transient’ and ‘resident’ animals in our study, a more detailed outline of habitat use and movements is being developed for animals that utilise our study region but may not nest in the local area. Although it appears that sub-adults are more ‘transient’ than mature animals, the small sample size means than further tracking and genetic analysis, targeting both adult and sub-adult animals, is required to gain a better understanding of their behavior.

Home ranges of turtles that remained in the Gulf were generally large, ranging between 529 and 5023 km^2^ with a mean of 1390 **±** 1783 (median = 683, n = 6). These home range estimates are likely to be impacted by the low accuracy of Argos locations and therefore, so are comparisons between individual animals and other studies. Broderick et al. [72] tagged 10 post-nesting green turtles in the Mediterranean and found turtles formed home ranges of less than 77 km^2^. Rees et al. [18] reported similar home ranges of below 76 km^2^. Of the resident turtles in this study, all home ranges were in excess of 500 km^2^, suggesting that turtles in the Gulf waters of the UAE utilize much larger areas. Schofield et al. [73] found that loggerheads in the Mediterranean dispersed widely, with females dispersing further than males. This extensive dispersal was attributed to the widespread availability of foraging grounds throughout the Mediterranean. The coastal waters of the UAE have suitable green turtle habitat spread along the entire Gulf coast which may similarly explain the large home range sizes.

Rees et al. [18] reported core habitat area from two rehabilitated turtles in Kuwaiti waters of the Gulf as 15 km^2^ and 8 km^2^, and from one post-nesting female at 5 km^2^ (mean = 9 ± 3). In comparison, the core habitat area for our resident turtles ranged between 8 km^2^ and 112 km^2^ with a mean of 50 ± 42 km^2^ (median = 56, n = 5), notably larger, although [18] reported that a low number of transmitted locations were used for the tracked nesting female which may have reduced the calculated core area size. Two adult females (‘Jade’ and ‘Belle’) formed small core home ranges (<16 km^2^) but three others (‘Bahar’, Emerald and ‘Moonlight’) formed larger core habitats (<112 km^2^) within the same local area between Dubai and Abu Dhabi. Four turtles, ‘Bahar’, ‘Moonlight’, ‘Belle’ and ‘Emerald’ formed home ranges within the Ghantoot area, known to have healthy sea grass beds and supporting a sizeable population of green sea turtles.

This wide-ranging availability of food within their home range may explain why core habitat area was large and why, during their tracked time, they did not venture far. Seminoff et al. [71] tracked 12 sub-adult and adult green turtles in Mexican waters, and reported kernel density home ranges with one to three activity centers or core habitats ranging in area from 0.038 to 6.42 km^2^ (mean = 1.788 ± 0.62 km^2^) it was reported that green turtles displayed small and defined home ranges which were dependent on the extent of suitable habitat and concluded that there was no evidence that size, sex and duration of tagging had an effect on the area utilised. Makowski et al. [74] tracked six juvenile green sea turtles from Atlantic waters around Florida, core areas from kernel density ranged from 0.18 and 1.17 km^2^ (mean = 0.49 ± 0.39 km^2^). Both studies have notably smaller core habitat areas than our resident turtles which may be attributed to a larger area of suitable and productive habitat along the UAE Gulf coastline.

### Thermoregulatory behavior

Observations by the DTRP of turtles kept in sea-fed outside enclosures over the summer months showed that when the water temperature reached 34°C, the smaller juvenile hawksbill turtles *E*. *imbricata* (<10kg) started to show signs of heat stress as displayed by lethargy and a reduction in feeding (DTRP, unpub. data). Once these turtles were relocated to cooler, temperature-controlled holding facilities, normal feeding and behaviour resumed. The heat stress symptoms were only displayed in juvenile hawksbills whilst larger individuals of the same species and of green turtles of all life stages did not display these symptoms even though the water during the summer months can exceed 36°C. These observations suggest that the upper thermal tolerance levels of juveniles may differ from sub-adults and adults in some turtle species. They also show that green sea turtles can tolerate very warm water. Spotila et al. [75] suggested that thermoregulatory capabilities in sea turtles increase with body size, which may explain why coastal areas are frequented by sub-adults and adults, while juveniles are rarely encountered. Mestre et al. [34] and [76] suggested that sea water temperature was a variable of minor importance in regard to the movement paths chosen by green turtles in the Atlantic Ocean including the Caribbean Sea. However, the Gulf is probably the warmest sea in the world [21] and turtles in this region are likely exposed to the highest seawater temperatures. Resident green turtles tagged and released during this study encountered water temperatures in excess of 36°C whilst within their coastal core habitats.

Pilcher et al. [77] reported a short-term behavioural response to remotely sensed Sea Surface Temperature (SST) in Gulf hawksbills. Their results suggest that hawksbills moved towards deeper and cooler waters in the summer months. We saw no such thermoregulatory movements away from the coast in the summer months using tag collected temperature data, indicating that the upper-temperature tolerance level for green turtles is more than 36°C. This apparent resilience to high water temperatures has further implications for studies on climate change and its effect on turtle behaviour in other regions as sea temperatures rise. Pilcher et al. [78] found no indication of extreme sex bias in both hawksbill and green turtle populations from Qatari waters of the Gulf, even with the extreme high temperatures experienced and it is suggested that marine turtles in the Gulf may be able to adapt to these warmer conditions. Although our study indicates that sub-adult and adult green turtles may display resilience to increasing sea temperatures, this may not be the case for all stages of their embryonic development and life cycle stages and further research is suggested.

### Conservation implications

Green turtles face many threats in UAE waters, including collisions with vessels, direct and incidental take, as well as habitat destruction and degradation from coastal development. The fate of Bahar in this study exemplifies the ongoing anthropogenic threats to wild turtles in UAE waters, even though all sea turtle species are protected under UAE law. Our results show that some coastal waters in the UAE are important habitats for green turtles throughout the year and as such, should be considered for further protection.

Rehabilitation efforts were successful, with sea turtles surviving in the wild after long periods of captivity. This provides support to the premise that rehabilitated animals are potential candidates for ecological tracking studies. Sea turtle tracking studies are heavily biased towards nesting females. Rehabilitated turtles offer the opportunity to track members of other demographic groups and therefore support our understanding of these species.

Four of the eight turtles remained in UAE waters over the period of tracking, highlighting the country’s critical role in managing and protecting green turtles and the habitats they utilize. However, movements across international maritime boundaries were frequent for released turtles and included movements into the waters of Iran, Pakistan, Oman, Maldives, Sri Lanka and India. Mitigation measures for threats they might face during their travels are crucial and require broad, cross-boundary and collaborative sea turtle management on a regional scale to ensure their effectiveness.

## Supporting information

S1 Fig An image of ‘Dibba’ being released from a beach in the UAE. (TIF)

S1 Data The individual post-filtered satellite transmitted data for each sea turtle included in this study. (XLS)
