# Supplementary figures and images for: Satellite tagging of rehabilitated green sea turtles Chelonia mydas from the United Arab Emirates, including the longest tracked journey for the species
Source: PLoS One. 2017 Sep 5;12(9):e0184286. doi: 10.1371/journal.pone.0184286 (PMC5584959; doi:10.1371/journal.pone.0184286)

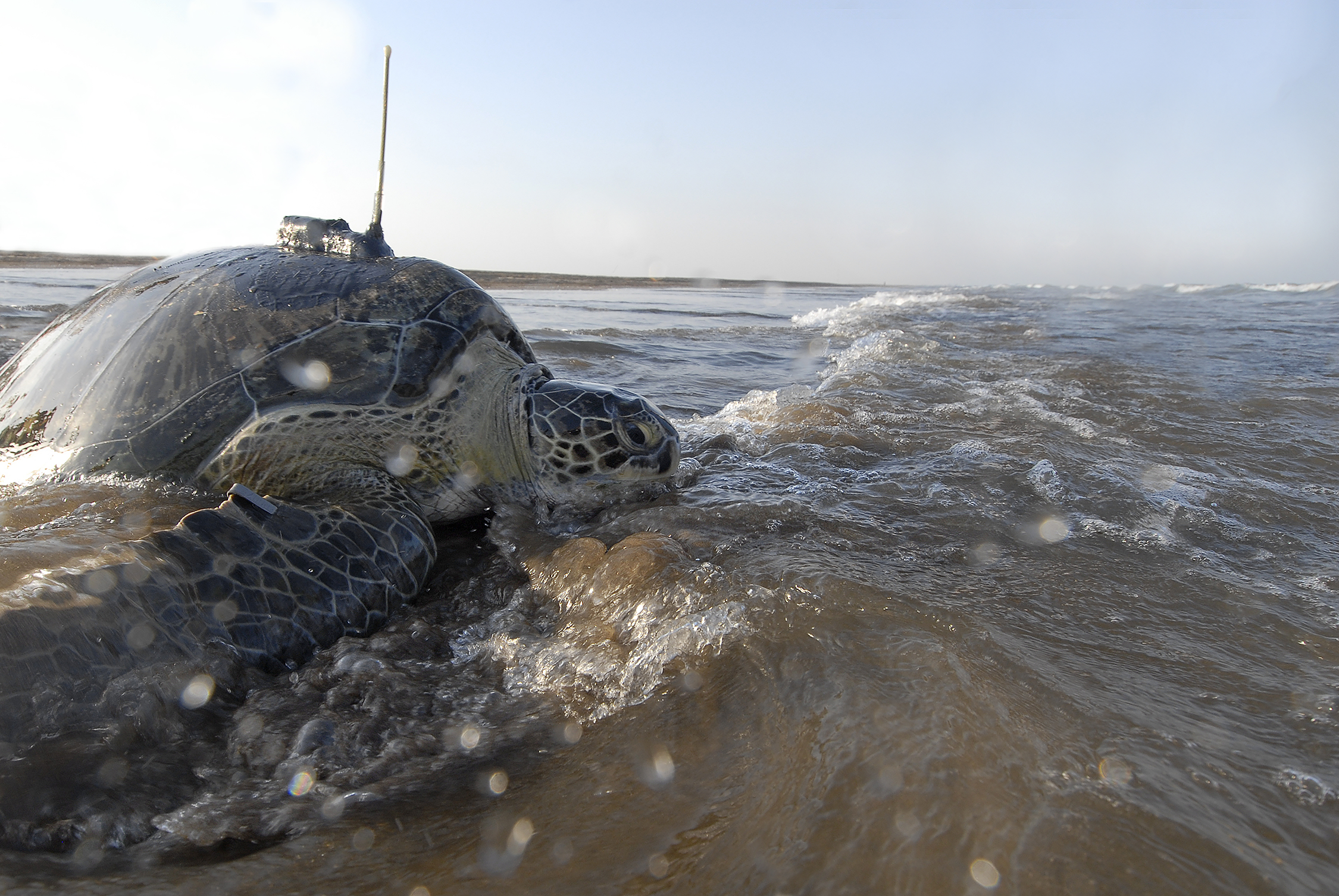

Supplement: S1 Fig — (TIF) [file pone.0184286.s001.tif]
